# Metformin and Colorectal Cancer

**DOI:** 10.3389/fendo.2018.00622

**Published:** 2018-10-23

**Authors:** Takuma Higurashi, Atsushi Nakajima

**Affiliations:** Department of Gastroenterology and Hepatology, Yokohama City University School of Medicine, Yokohama, Japan

**Keywords:** metfromin, colorectal cancer, chemoprevention, epidemiology, basic research, review, clinical trials

## Abstract

Colorectal Cancer (CRC) is one of the most frequently encountered neoplasms in humans. The incidence of CRC has been increasing and new strategies for prevention, including chemoprevention, are required to lower its incidence and associated mortality. Metformin is a biguanide compound commonly used for the treatment of diabetes mellitus. Many recent basic research, epidemiological and clinical trial studies have indicated that metformin has benefits not only in diabetes treatment, but also in lowering the risk of developing cancer (including CRC). These studies indicate that metformin may be a candidate chemoprevention agent for CRC. This review article shall discuss the present evidence of metformin treatment and CRC, as well as outline our challenge in the investigation of metformin use in chemoprevention therapy for colorectal tumors.

## Introduction

Colorectal Cancer (CRC) is one of the most frequently encountered neoplasms across the world. The incidence of CRC has rising in many low- and middle-income countries, and some highly-developed countries (1). Despite great advances in cancer treatment over the last two decades, such as the development of more effective drugs with improved safety and more precise molecular targeting, unwanted adverse effects remain a major problem. New cancer treatments are also extremely expensive. The prevention or reduced incidence of cancer would help lower rising medical costs (2), providing a cheaper and more effective strategy of decreasing cancer mortality. The resection of colorectal polyps lowers the risk of future development of advanced adenoma and CRC (3). Yet patients with polyps (adenomas and/or hyperplastic polyps) remain at high risk for the development of future colorectal polyps and CRC (4). This ongoing risk highlights the need for a conceptual change, from surveillance and detection of adenomas and cancer (the former often being treated by endoscopic resection) to new strategies for prevention, including chemoprevention, to lower the incidence and associated mortality of CRC.

A number of agents have been reported to have a chemopreventive effect against colorectal carcinogenesis. In regard to epidemiology, the 2011 World Cancer Research Fund and American Institute for Cancer Research reported beneficial food and nutrition for decreasing the incidence of CRC (5) (Table 1). However, effective clinical trials have been limited. Nonsteroidal anti-inflammatory drugs (NSAIDs), notably cyclooxygenase-2 (COX-2) inhibitors, used either alone or in combination with other agents, have offered the most potential for lowering the risk of CRC. Unfortunately, there is an elevated risk of serious cardiovascular events associated with the administration of COX-2 inhibitors (6, 7). Considering these cardiovascular side effects and the lack of demonstrable efficacy of other drugs that initially showed potential in this setting, novel agents are required that are clinically effective and safe for CRC prevention. An increased incidence of CRC in adults is also associated with obesity and diabetes mellitus (8, 9). Therefore, we predicted that these conditions may provide novel targets for the chemoprevention of CRC.

**Table 1:** Foods and nutrients with supporting findings in epidemiological studies.

**Convincing**	**None**
Probable	Food containing dietary fiber
	Garlic
	Milk
	Calcium
Limited-suggestive	Non-starch vegetables
	Fruits
	Food containing folate
	Fish
	Food containing selenium
	Food containing vitamin D
	Selenium

Many recent reports, including basic research, epidemiological and clinical trial studies, suggested that metformin also lowered the risk of developing malignant disease, such as CRC. Accumulating data indicates that metformin may be a candidate chemoprevention agent for CRC. We shall discuss the current evidence of metformin administration and CRC risk and outline our challenge of using metformin for the chemoprevention of colorectal tumors.

## Metformin and colorectal cancer

### Epidemiological research

The first report of a relationship between metformin administration and the risk of CRC was published in 2004 (10). Subsequently, many population-based and case-control cohort studies, and associated meta-analysis, have evaluated metformin use and the risk of CRC. Different studies reported a decreased risk (11–17), no association (18–21), or an increased risk of CRC (22, 23). The reason for different conclusions between certain studies may be related to time-related biases, which were proposed to account for some of the inverse associations observed between metformin administration and cancer risk reported in epidemiologic studies (24, 25). These include immortal time bias when unexposed time is misclassified, as in cohort studies, time-window bias when the time window for capturing exposure differs between cases and controls in case/control studies, or time-lag bias when treatment differs across stages of the disease (with disease stage also associated with the outcome risk). A recent cohort study that minimized these biases concluded there was an inverse association between long-term administration of metformin and CRC risk (26). Further studies and detailed analyses are needed to clarify the potential clinical benefits of metformin upon the incidence and associated mortality of CRC.

### Basic research

In preclinical research, metformin suppressed cell proliferation, increased apoptosis, caused cell cycle arrest, and suppressed the incidence and growth of experimental tumors *in vitro* and *in vivo* (27–29). The underlying molecular mechanism of metformin action was shown to involve liver kinase B1 (LKB-1)-dependent activation of AMP-activated protein kinase (AMPK) (30, 31). Molecular mechanisms of metformin actions were mostly studied in adipose and liver tissue in relation to glucose homeostasis and insulin actions. Recent studies reported involvement of the AMPK/mammalian target of rapamycin (mTOR) pathway in the induction of various cancers (32, 33). Downstream targets of mTOR signaling include proteins that control translational machinery, including the ribosomal protein S6 kinases (S6K) that regulate the initiation and elongation phases of translation (34). The upstream regulation of mTOR involves signaling pathways of several oncoproteins or tumor suppressors, including AMPK, phosphatidyl inositol 3-kinase and phosphatase and tensin homolog (35). In particular, upregulation of AMPK directly suppresses mTOR, resulting in the inhibition of cell proliferation (36). In addition, *in vitro* analysis demonstrated that the metformin-induced suppression of the growth of breast cancer cells was associated with decreased activation of mTOR and S6 kinase (37).

The above findings indicated that metformin was effective at reducing carcinogenesis *in vitro*. We will now focus on reported *in vivo* experiments and our study of colon carcinogenesis using several animals models. The first report of phenformin that inhibit metabolic immunodepression in rats 1977 (38). From then, several reports showed that biguanide prevent colon carcinogenesis. Experimental rodent models of CRC can be broadly separated into genetic (such as *Apc*^Min/+^ mice, a murine model of familial adenomatous polyposis coli (APC)) and chemical carcinogen-induced (such as azoxymethane (AOM)-induced) sporadic models. Many studies of chemoprevention have used both rodent models of CRC, however, some studies reported that candidate agents had consistent preventive effects in both models, whereas other studies reported inconsistent and contradictory results (39). Therefore, it is important to investigate the ability of candidate chemoprevention agents to suppress tumorigenesis in both the genetic and sporadic cancer models. First, we examined the effect of metformin on intestinal polyp growth in *Apc*^Min/+^ mice. Nine-week-old *Apc*^Min/+^ mice were split into two groups: one received metformin (250 mg/kg per day in the diet) treatment, the other received a normal diet without metformin, and the number and size of polyps were analyzed in both groups after 10 weeks. Administration of metformin significantly suppressed the number of intestinal large polyps formed in *Apc*^Min/+^ mice (40). Second, we investigated a carcinogen-induced sporadic colorectal cancer model. Seven-week-old mice were administered AOM by intraperitoneal injection and then treated with or without metformin for 6 weeks (to investigate aberrant crypt foci (ACF) formation) or 32 weeks (for tumor formation). Metformin treatment significantly inhibited ACF and polyp formation. Furthermore, western blot analysis showed that metformin treatment stimulated AMPK phosphorylation, and significantly inhibited the phosphorylation of mTOR, S6K and S6 proteins. It was proposed that metformin suppressed colonic mucosal proliferation via activation of AMPK and then the downstream suppression of the mTOR pathway (41). In other animal model, it has been shown that metformin dosedependently inhibits the development of colon tumors induced by 1,2dimethylhydrazine (DMH) in rats (42, 43). In this way, many reports showed that metformin is effective for colorectal carcinogenesis both *in vivo* and *in vitro*.

### Clinical trials

**Figure 1:**
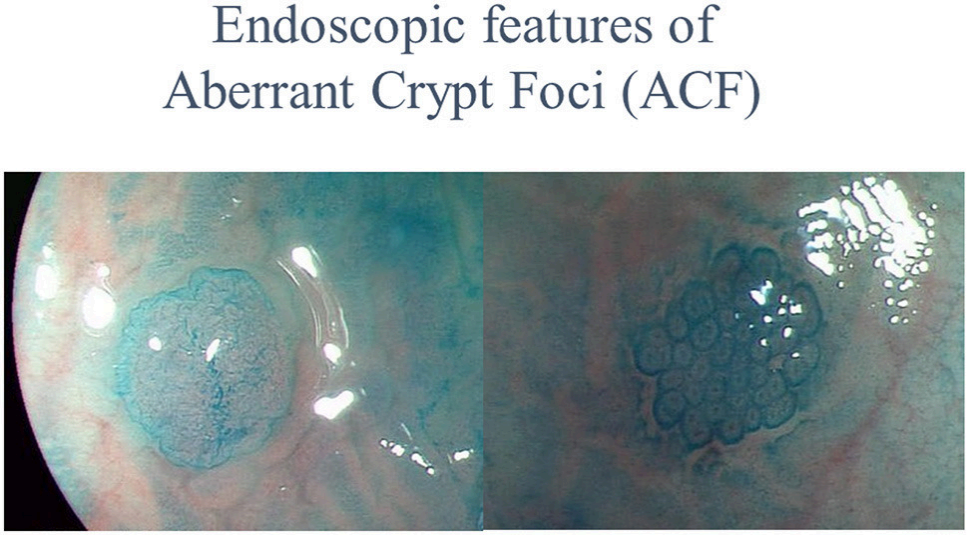
Endoscopic features of Aberrant Crypt Foci (ACF).

Previous basic research and epidemiological studies indicated that metformin had a chemopreventive effect upon CRC. However, confirmation of metformin efficacy required a prospective interventional trial. In chemoprevention trials targeting CRC, the incidence of adenomas or the cancer itself was generally used as the main endpoint. While the occurrence of CRC is a clear endpoint, its low incidence in the general population, and the required long-term observational period make this endpoint unsuitable for chemoprevention trials (44). The use of surrogate biomarkers for cancer detection may allow evaluation of drug efficacy in a shorter timeframe. Aberrant crypt foci are very small lesions that develop in the earliest stage of colorectal carcinogenesis, and consist of large, thick crypts that can be detected by dense methylene blue staining (45–47), as shown in Figure 1. The ACF were reported to be precursor lesions for human colorectal carcinogenesis (48), and were proposed as a surrogate endpoint in chemoprevention trials for CRC. Several studies have examined the correlation between the presence and number of ACF and use of candidate chemopreventive agents for CRC in humans. The presence and number of ACF were found to be suppressed by certain chemopreventive agents (49, 50). There are several advantages to using colorectal ACF as the primary endpoint in CRC chemoprevention trials. First, a long-term observational period is not needed to evaluate agent effects; thus avoiding long-term trials, which require considerable effort and may expose trial participants to an increased risk of carcinoma occurrence. Second, ACF can be estimated quantitatively. In 2010, there were no reported prospective metformin chemoprevention trials, so we implemented a pilot prospective clinical trial to examine the efficacy and safety of metformin use and its effects upon ACF formation. We prospectively randomized 26 participants with colorectal ACF to receive treatment with metformin (250 mg/d) or no treatment, followed by evaluation of the number of ACF. Magnifying colonoscopy was used to determine the number of rectal ACF and other laboratory endpoints (using blind analysis) in each patient at baseline and after 1 month of treatment. Prior to treatment, there were no significant differences in the number of rectal ACF and other baseline clinical characteristics between the two groups. At 1 month, the mean number of ACF per patient was significantly reduced in the metformin group (8.78 ± 6.45 before treatment vs. 5.11 ± 4.99 at 1 month, *P* = 0.007), whereas the mean ACF number was unchanged in the control group (7.23 ± 6.65 vs. 7.56 ± 6.75, *P* = 0.609). This initial trial provided preliminary data suggesting that metformin inhibited human rectal ACF formation (51). However, this prospective trial had some limitations. First, the trial duration was only 1 month. Second, although ACF were considered a convenient surrogate biomarker of colorectal carcinogenesis (48), their biological significance remains controversial. Generally, the occurrence of CRC would be the most reliable endpoint in chemoprevention trials for CRC. However, there would be serious ethical issues in withholding endoscopic removal when resectable lesions (that develop into cancer) were detected in annual colonoscopies. In previous CRC chemoprevention trials, such as those investigating NSAIDs and aspirin, detection of the metachronous adenoma was set as the primary endpoint. Therefore, we used metachronous colorectal adenomas/polyps as the endpoint in our subsequent metformin chemoprevention trial. Previous CRC chemoprevention trials also involved initial short-term trials to establish safety and efficacy, followed by expanded trials of longer duration. Long-term trials require a large amount of resources and may expose the study participants to the risk of cancer. There have been no reported randomized control trials for CRC chemoprevention using metformin, and the safety of subjects would need careful attention in the design and execution of such a trial. Considering these issues, we designed a 1-year clinical trial to evaluate the safety and chemopreventive effect of metformin on sporadic CRC in patients at high risk of adenoma recurrence, as a preliminary study before considering long-term CRC chemoprevention trials. The trial protocol was previously published (52).

**Figure 2:**
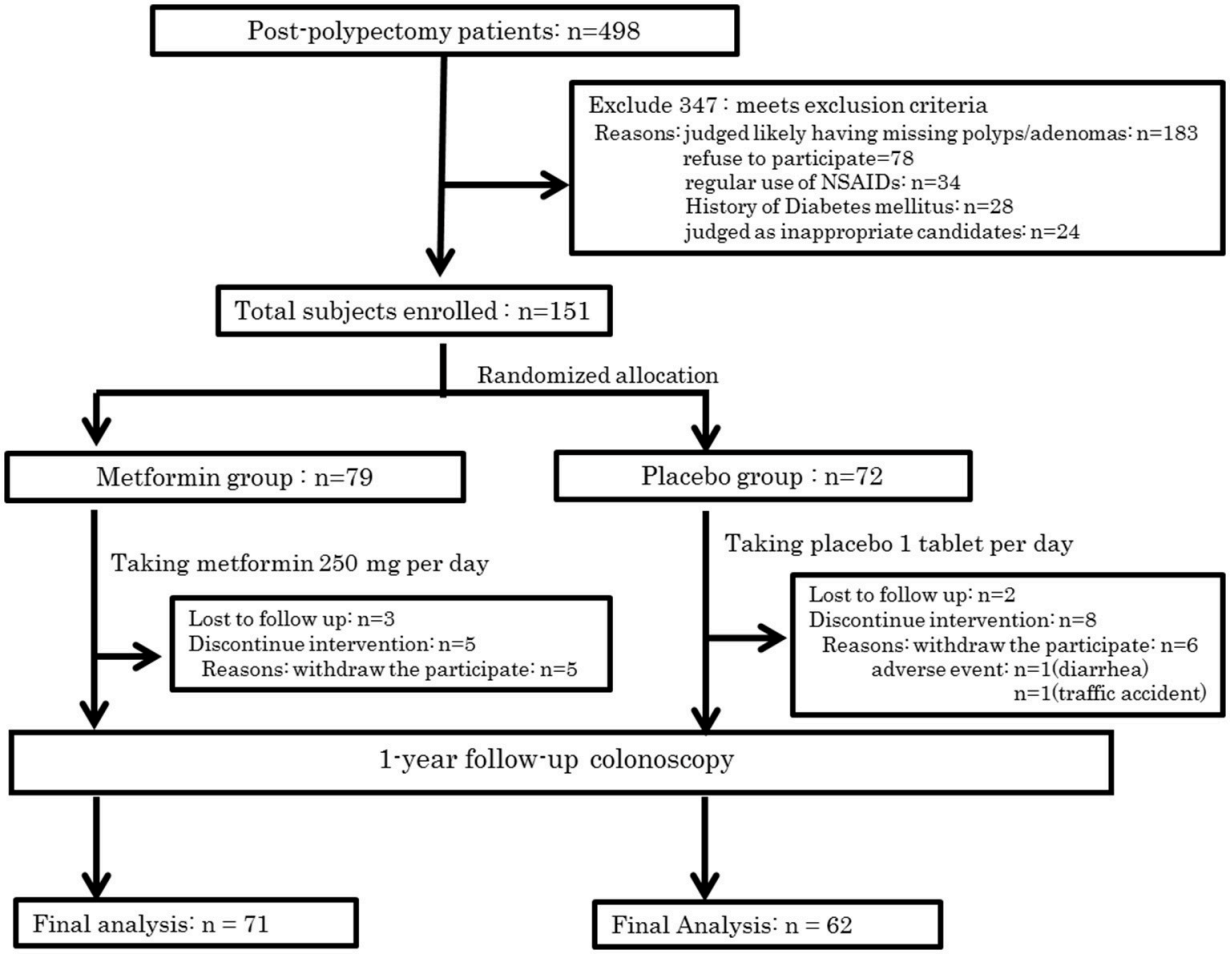
Trial profile. This figure is reproduced from (53) with permission.

In all, 498 subjects were screened for eligibility, and 347 subjects were excluded for the reasons shown in Figure 1. Of these participants, 183 cases were excluded owing to inadequate colon cleaning, such as an incompletely cleaned polypectomy, poor bowel preparation, short observation time or lack of insertion to caecum (the major reason was incomplete polypectomy). The 151 eligible patients were randomly allocated into two groups; 79 and 72 in the metformin and placebo groups, respectively (Figure 2). Of these 151 patients, five were lost to follow-up (three in the metformin group, two in placebo the group) and 13 withdrew their informed consent during the follow-up period. The remaining 133 patients (71 and 62 in the metformin and placebo groups, respectively) received a 1-year follow-up colonoscopy. Table 2 shows the baseline characteristics of the subjects. There were no diabetes mellitus patients in either group (exclusion criteria). In both groups, the proportion of subjects with advanced adenoma (including early carcinoma) and multiple adenomas was approximately 70%. The incidence of total polyps (adenomas plus hyperplastic polyps) in the metformin group was significantly lower than that in the placebo group [metformin group had 27/71, 38.0% (95% confidence interval (CI), 26.7–49.3) vs. the placebo group with 35/62, 56.5% (95% CI, 44.1–68.8); *p* = 0.034]. The risk ratio (RR) was 0.674: 95% CI, 0.466–0.974. The incidence of adenomas in the metformin group was also significantly lower than that in the placebo group [metformin group had 22/71, 30.6% (95% CI, 19.9–41.2) vs. the placebo group with 32/62, 51.6% (95% CI, 39.2–64.1), *p* = 0.016]. The RR was 0.600 (95% CI, 0.393–0.916; Table 3). The incidence of adverse events was approximately 10% and equivalent between the two groups (Table 4). All adverse events were considered very mild, such as abdominal pain, diarrhea, and exanthema.

**Table 2:** Baseline characteristics of the subjects.

	**Metformin**	**Placebo**
No of subject	71	62
Age, (mean ± SD), y	63.1 ± 8.5	63.5 ± 10.2
Sex (M/F)	54/17	49/13
BMI	23.1 ± 2.6	23.9 ± 3.5
Family history of CRC	8 (11%)	10 (16%)
Current smoker	23 (32%)	25 (40%)
History of Diabetes	0	0
History of Hyperlipidemia	15 (21%)	7 (11%)
History of Hypertension	20 (28%)	20 (32%)
Finding of baseline CS		
Multiple & Advanced adenoma + early carcinoma	51 (72%)	43 (69%)

*This table is reproduced from (53) with permission*.

*CS, colonoscopy; Multiple, more than 3 adenomas; Advanced adenomas, high-grade dysplasia, large size (>10 mm), or villous features*.

**Table 3:** Incidence of total polyps and adenomas 1 year after the start of treatment.

	**Metformin**	**Placebo**	***p*-value**
Incidence of total polyp (95%CI) Risk ratio (95%CI)	27/71 (38.0%) (26.7–49.3) 0.674 (0.466–0.974)	35/62 (56.5%) (44.1–68.8) 1(reference)	0.034
Incidence of total adenomas (95%CI) Risk ratio (95%CI)	22/71 (30.6%) (19.9–41.2) 0.600 (0.393–0.916)	32/62 (51.6%) (39.2–64.1) 1 (reference)	0.016

*This table is reproduced from (53) with permission*.

*CI, confidence interval; IQR, interquartile range*.

**Table 4:** Adverse events in the metformin and placebo groups.

**Adverse events**	**Metformin**	**Placebo**
Abdominal pain	0	1
Diarrhea	1	4
Rash	2	0
Constipation	3	3
Alopecia	0	1
Total	6	9

*This table is reproduced from (53) with permission*.

*All adverse events were NCI-CTCAE grade 1*.

This study was the first clinical trial to examine the chemoprevention effect of low-dose metformin on metachronous colorectal adenoma/polyp formation. Metformin was shown to suppress metachronous colorectal adenoma/polyp formation (53). This clinical trial had possible limitations. First, the follow-up colonoscopy at 1 year may be too soon, because many chemoprevention trials for metachronous adenoma formation had used study durations of 3 years to 5 years. However, no previous metformin chemoprevention trials were reported, and a trial longer than 1 year may present ethical concerns. In an attempt to overcome these issues, we choose participants who were at high risk of adenoma and cancer occurrence. Patients who have had multiple and advanced adenomas (high-grade dysplasia, large adenomas >10 mm, and villous features) are known to be at high risk of CRC (3), and surveillance after endoscopic resection is recommended for up to 3 years (54). In the current trial, almost 70% of subjects in each group had previously exhibited advanced adenoma (including early carcinoma) or multiple adenomas. However, long-term observation of post-polypectomy patients, a high-risk group for CRC, may entail ethical problems. Placebo group subjects who received resection of advanced or multiple adenomas showed a high rate (30/43, 70%) of recurrence, and this rate was a little higher than that found in previous chemoprevention trials for adenoma recurrence. However, there was no CRC detected in any subjects in the 1 year follow-up colonoscopy. To validate the efficacy of metformin for the prevention of CRC, further long-term studies are needed. The second limitation is that the trial did not study dose–response effects of metformin on metachronous colorectal adenomas/polyps. Previous trials of metformin for cancer prevention and adjuvant treatment have been conducted using high-doses of metformin (500–2,000 mg/day). Unfortunately, high-dose metformin is associated with an increased risk of developing lactic acidosis and adverse gastrointestinal effects, such as diarrhea. Gontier et al. reported a PET/CT trial in which subjects received medication with anti-diabetic drugs, including metformin, and exhibited high and diffuse intestinal uptake of ^18^F-fluorodeoxyglucose (55). This finding indicates that AMPK is abundant in intestinal mucosa and that activation of AMPK by metformin up-regulates the expression of glucose transporters. Therefore, metformin-induced chemoprevention in the colorectum appears to be a reasonable strategy targeting key molecular pathways. In a previous study, we found that oral low-dose metformin (250 mg/day) was safe and inhibited human colorectal ACF and metachronous adenoma formation (51, 53). We predict that oral low-dose metformin also has clinical efficacy for CRC chemoprevention. The third limitation of this study was that many participants in this trial were at high risk of adenoma and cancer recurrence. Around 70% of participants had advanced and multiple adenomas (or early carcinoma). This proportion was high compared with other chemoprevention trials. However, because of the randomization process, there was no internal bias in the groups. Nevertheless, our trial did not directly determine the efficacy of metformin for patients with an average risk of CRC (external validity). Finally, this trial was conducted in a small region of Japan and the sample size was small. Many previous adenoma prevention trials, including that of celecoxib, were carried out in Western countries. Future well-designed clinical chemoprevention trails are required that include larger sample sizes and involve many multinational institutions and more ethnic groups.

## Conclusion and future perspective

A practical chemoprevention agent generally requires the following attributes: safety, good compliance, cost effectiveness, and a clear mechanism. Metformin meets these criteria. To date, NSAIDs, especially COX-2 inhibitors, have provided the most reliable risk reduction for CRC, but they also confer an increased risk of severe cardiovascular events (6, 7). Metformin, first synthesized in the 1920s, has been used worldwide for treating diabetes mellitus, metabolic syndrome and polycystic ovary syndrome (56). In the present clinical study, the use of low-dose metformin for 1 year caused few adverse events, which were all very mild. These findings indicate that low-dose metformin is safe. In addition, metformin is an inexpensive medicine suitable for daily use. Generally, patients need chemopreventive agents as a long term therapy. Metformin is suitable in these conditions. Finally, the mechanism of action has been well elucidated for metformin. Metformin is known to activate AMPK, which inhibits the mTOR pathway that plays an important role in cellular translational processes and progression (30). Although more than 100 randomized controlled trials of metformin and cancer are currently registered at ClinicalTrials.gov, the vast majority are testing the effect of metformin in cancer treatment rather than prevention. This situation perhaps underscores the inherent challenge of doing chemoprevention trials with cancer endpoints, which mandate follow-up of many individuals over many years. As an efficient and feasible alternative, trials designed to examine the effect of metformin on cancer biomarkers or surrogate endpoints over a shorter time horizon are an important next step before embarking on expensive larger scale trials (57). For colorectal cancer prevention specifically, a large-scale randomized controlled trial of metformin (perhaps in combination with aspirin, an established chemopreventive agent) for adenoma recurrence in a population with a broader risk profile appears warranted (58).

In conclusion, metformin has the potential to provide a novel chemoprevention therapy for CRC. However, to fully clarify the chemopreventive effect of metformin on CRC, further large-sample size and long-term clinical trials are required.

## Author contributions

TH and AN conceived the study. All the authors have read the final manuscript and approved its submission for publication.

### Conflict of interest statement

The authors declare that the research was conducted in the absence of any commercial or financial relationships that could be construed as a potential conflict of interest.
